# Long‐term miR‐29b suppression reduces aneurysm formation in a Marfan mouse model

**DOI:** 10.14814/phy2.13257

**Published:** 2017-04-28

**Authors:** Homare Okamura, Fabian Emrich, Jeffrey Trojan, Peter Chiu, Alex R. Dalal, Mamoru Arakawa, Tetsuya Sato, Kiril Penov, Tiffany Koyano, Albert Pedroza, Andrew J. Connolly, Marlene Rabinovitch, Cristina Alvira, Michael P. Fischbein

**Affiliations:** ^1^ Department of Cardiothoracic SurgeryStanford UniversityStanfordCalifornia; ^2^ Department of PathologyStanford UniversityStanfordCalifornia; ^3^ Department of PediatricsStanford UniversityStanfordCalifornia

**Keywords:** Aneurysms, aorta, Marfan syndrome, microRNA

## Abstract

Aortic root aneurysm formation and subsequent dissection and/or rupture remain the leading cause of death in patients with Marfan syndrome. Our laboratory has reported that miR‐29b participates in aortic root/ascending aorta extracellular matrix remodeling during early aneurysm formation in *Fbn1* ^C1039G/+^ Marfan mice. Herein, we sought to determine whether miR‐29b suppression can reduce aneurysm formation long‐term. *Fbn1* ^C1039G/+^ Marfan mice were treated with retro‐orbital LNA‐anti‐miR‐29b inhibitor or scrambled‐control‐miR before aneurysms develop either (1) a single dose prenatally (pregnant *Fbn1* ^*C1039G/+*^ mice at 14.5 days post‐coitum) (*n* = 8–10, each group) or (2) postnatally every other week, from 2 to 22 weeks of age, and sacrificed at 24 weeks (*n* = 8–10, each group). To determine if miR‐29b blockade was beneficial even after aneurysms develop, a third group of animals were treated every other week, starting at 8 weeks of age, until sacrificed (*n* = 4–6, each group). miR‐29b inhibition resulted in aneurysm reduction, increased elastogenesis, decreased matrix metalloproteinase activity and decreased elastin breakdown. Prenatal LNA‐anti‐miR‐29b inhibitor treatment decreased aneurysm formation up to age 32 weeks, whereas postnatal treatment was effective up to 16 weeks. miR‐29b blockade did not slow aortic growth once aneurysms already developed. Systemic miR‐29b inhibition significantly reduces aneurysm development long‐term in a Marfan mouse model. Drug administration during aortic wall embryologic development appears fundamental. miR‐29b suppression could be a potential therapeutic target for reducing aneurysm formation in Marfan syndrome patients.

## Introduction

Patients with Marfan syndrome (MFS) typically develop aortic root aneurysms with ensuing aortic dissection remaining the leading cause of death (Judge and Dietz 2005). Over the last decade, numerous studies have demonstrated that the underlying fibrillin‐1 gene mutation in MFS increases the activity of transforming growth factor‐*β* (TGF‐*β*) (Dietz 2010; Neptune et al. 2003). Although TGF‐*β* blockade inhibits aneurysm formation and growth in murine models of MFS, the molecular mechanism by which excessive TGF‐*β* signaling leads to aneurysm development remains unknown. A better understanding behind the pathophysiology of aneurysm formation will result in novel early detection methods and potentially new treatment options.

MicroRNAs (miRNA) are endogenous small single‐stranded, noncoding RNA molecules that repress gene expression by partial or complete complementary binding to target sequences within mRNA (Chitwood and Timmermans 2007; Mishra et al. 2009). Our laboratory has identified that the miRNA, miR‐29b plays an important pathogenic role in early aneurysm development in a Marfan mouse model *(Fbn1* ^C1039G/+^). MiR‐29b is increased during early postnatal development exclusively in the *Fbn1* ^C1039G/+^ aortic root/ascending (AS) aorta, correlating with the clinical scenario observed in MFS patients, where aneurysms typically form in the aortic root. Moreover, miR‐29b suppression by anti‐sense oligonucleotides prevented aneurysm development at age 4 weeks and precludes aortic wall apoptosis and extracellular matrix (ECM) remodeling (Merk et al. 2012). However, whether miR‐29b blockade results in durable protection against aneurysm formation is not known.

The miR‐29 family has been reported to promote ECM remodeling through its putative gene targets, including collagen, elastin, fibrillin‐1, and matrix metalloproteinases (MMP) 2 and 9 (Chen et al. 2011; van Rooij et al. 2008). Interestingly, their role in aneurysm development is controversial and may depend on species, embryologic origin, location of aneurysm, and timing. Although we reported that miR‐29b was overexpressed during early AS aortic aneurysm formation in a Marfan mouse model, Maegdefessel et al. found reduced miR‐29b expression during abdominal aortic aneurysm (AAA) development in two murine experimental models (Maegdefessel et al. 2012; Merk et al. 2012). In addition, while in vivo administration of locked nucleic acid (LNA) anti‐miR‐29b (retro‐orbital) reduced aneurysm formation in both animal model systems, it was associated with reduced elastin breakdown in the Marfan model system versus increased collagen deposition and fibrosis in the AAA models (Maegdefessel et al. 2012; Merk et al. 2012). In the present study, using the *Fbn1* ^C1039G/+^ Marfan mouse model, we illustrate that miR‐29b blockade with an oligonucleotide inhibitor slows aneurysm growth long‐term. Intriguingly, a single dose of prenatal (in utero) miR‐29b inhibitor is superior to a continuous postnatal regimen, suggesting that the pathogenesis of aneurysm formation in Marfan mice may be initiated during early embryologic development.

## Materials and Methods

### Mice

Both male and female heterozygous *Fbn1* ^C1039G/+^ mice and C57BL/6J littermate wild‐type (WT) controls were used. In order to inhibit miR‐29b, animals were treated with either a LNA‐anti‐miR‐29b inhibitor (mmu‐miR‐29b, miRCURY LNA^TM^ microRNA Power inhibitor, Exiqon, Woburn, MA) or scrambled‐control‐miR using the following three different regimens:

1. *Prenatal LNA treatment*. A single retro‐orbital injection was administered to pregnant *Fbn1* ^*C1039G/+*^ mice at 14.5 days post‐coitum with either (1) LNA‐anti‐miR‐29b (5′‐3′ sequences ACTGATTTCAAATGGTGCT); or (2) scrambled‐control‐miR (5′‐3′ sequences GTGTAACACGTCTATACGCCCA) (miRCURY LNA microRNA inhibitor from Exiqon).
2. *Postnatal LNA treatment*. Two‐week‐old *Fbn1* ^C1039G/+^ mice received a retro‐orbital injection of (1) LNA‐anti‐miR‐29b or (2) scrambled‐control‐miR every other week for 22 weeks and sacrificed at 24 weeks.
3. *Late LNA treatment*. Following aneurysm formation, we treated *Fbn1* ^C1039G/+^ mice with a retro‐orbital injection of (1) LNA‐anti‐miR‐29b or (2) scrambled‐control‐miR at 8 weeks of age. Mice were treated every other week up to 24 weeks.

The dosage of anti‐miR or scrambled miR was 4 mg/kg in all treatment groups. The optimal dose was determined with a titration curve.

All animal procedures and experiments were approved by the Administrative Panel on Laboratory Animal Care at Stanford University. All experiments were carried out in accordance with the NIH and USDA Guidelines for the Care and Use of Animals in Research.

### Echocardiography

To assess AS aortic diameter, B‐mode transthoracic echocardiography (TTE) was performed at 4, 8, 16, 24 and 32 weeks**.** Mice were sedated with 2% inhaled isoflurane (2‐chloro‐2‐(difluoromethoxy)‐1,1,1‐trifluoro‐ethane) (Baxter Healthcare Corporation, Deerfield, IL) during the TTE procedure. The AS aortic diameter was measured in the parasternal long axis view (outer edge to outer edge) using a Vevo‐770 (Visualsonics, Toronto, Canada). Mice from the prenatal LNA treatment group were sacrificed at 32 weeks. The postnatal treatment group was sacrificed at 24 weeks, the time point when aneurysm formation did not differ significantly from that of *Fbn1* ^C1039G/+^ mice treated with scrambled‐control‐miR. Echo measurements were repeated three times and performed by two blinded investigators.

### Elastin Verhoeff's Van Gieson staining

The AS aorta was dissected and fixed in 4% paraformaldehyde. The aortic tissue was embedded in OCT Compound Histomount (Sakura, Torrance, CA) and cut into 4 *μ*m cross sections. The slides were stained with Accustain Elastin Verhoeff's Van Gieson (EVG) kit (Sigma‐Aldrich, St. Louis, MO) and imaged at 40× magnification using Leica DM4000B. The elastic lamina in three AS aortic sections per slide were assessed by a blinded pathologist for: (a) average number of breaks per elastic lamina by counting them circumferentially in all lamina; (b) EMi‐quantitative assessment of elastic lamina thinning on a scale of: 0 = none, 1 = mild; 2 = moderate; 3 = severe (i.e., to the point of multi‐perforate in areas). Experiments included four mice per group, using three consecutive sections of the AS aorta from each animal.

### Picrosirius red staining

Samples were stained using a Picrosirius Red stain kit (American MasterTech, Lodi, CA) according to manufacturer's instructions to assess for collagen. In summary, nuclei were stained with modified Mayer's hematoxylin. Samples were then rinsed in running tap water for 10 min, stained in Picrosirius Red for 1 h, washed in 0.5% acetic acid water, dehydrated in 100% ethanol, cleared in xylene, and mounted using a resinous medium. Aortic wall collagen was analyzed by polarized microscopy and quantified with Image J software. Experiments included five mice per group, using three consecutive AS aortic sections from each animal.

### In situ zymography for MMP activity

The AS aorta was harvested, embedded in OCT media without fixation and cut into 10 *μ*m serial sections. Low‐melting agarose (1%) (Sigma‐Aldrich, St. Louis, MO) was dissolved in phosphate‐buffered saline (PBS). Dye‐quenched gelatin (DQ‐gelatin) (1 mg/mL) (Invitrogen, Carlsbad, CA) was diluted 1:10 in low‐melting agarose solution. Agarose solution with or without DQ‐gelatin (200 *μ*L) was then placed on the aortic tissue sections and covered with a 22 × 40 mm glass coverslip. The sections were placed at 4°C for 30 min to make the agarose solid, then subsequently incubated at 37°C for 24 h. Specimens were visualized under fluorescent light at an excitation wavelength of 480 nm, with an exposure time of 50 msec. MMP activity was measured by calculating the difference in fluorescence between DQ‐gelatin‐containing sections and autofluorescence in sections without DQ‐gelatin (*n* = 3–5 in each group). Specimens were quantified with Image J software and the value of three sections per mouse was averaged.

### In situ zymography for non‐MMP activity

The AS aorta was harvested, embedded in OCT, stored at −80°C, and cut into 10 *μ*m serial sections. Low‐melting agarose (1%) (Sigma‐Aldrich, St. Louis, MO) was dissolved in assay buffer. DQ‐gelatin (1 mg/mL) (Invitrogen, Carlsbad, CA) was diluted 1:10 in low‐melting agarose solution. Increasing concentrations (0, 20, 50, 100, 200, and 300 mmol/L) of ethylenediaminetetraacetic acid (EDTA), a pan‐MMP inhibitor, was placed on the aortic specimens. We cannot exclude the possibility that EDTA cross‐reacts and blocks other matrix degrading enzymes. The sections were initially placed at 4°C for 30 min to allow the agarose to solidify, then incubated at 37°C for 24 h. The specimens were then imaged using a fluorescence microscope at an excitation wavelength of 480 nm, at 50 or 200× magnification. Autofluorescence sections were imaged at an exposure time of 20 msec.

### Elastase susceptibility test

The AS aorta was dissected and divided into two specimens longitudinally. Each sample was treated with PBS containing 0.5 U/mL of porcine pancreatic elastase (Invitrogen, Carlsbad, CA) for 20 min at 37°C. For controls, the elastase solution was heat‐inactivated (100°C) before use. Specimens were fixed in 4% paraformaldehyde for an hour. The aortic tissues were embedded in OCT Compound Histomount (Sakura, Torrance, CA) and cut into 7 *μ*m cross sections. The slides were mounted in glycerol on glass slides and then visualized with a Leica SP2 confocal system (Leica Microsystems, Wetzlar, Germany). Autofluorescence of elastin was excited at 488 nm, and emission was detected at 500–560 nm wavelengths. For semiquantification, the discontinuity score from 0 to 2 (0: normal, 1: 1–2 fold increase in elastic lamellar fenestration size; 2: 2–4 fold increase in elastic lamellar fenestration size) was assessed from five images in each sample by a pathologist blinded to genotype and treatment arm.

### Tissue RNA extraction and quantitative polymerase chain reaction

Total RNA was isolated using TRIzol reagent (TRIzol, Invitrogen, Carlsbad, CA). RNA was quantified by Nanodrop analysis (Agilent, Foster City, CA) and samples were included when the 260/280 nm ratio was >1.6. Diluted RNA was reverse‐transcribed using the TaqMan microRNA Reverse Transcription kit (Applied Biosystems, Life Technologies Carlsbad, CA) according to the manufacturer's instructions. Subsequently, the cDNA was amplified with the Viia 7 Real‐time PCR system with primers (Applied Biosystems, Life Technologies Carlsbad, CA). Gene expression levels were standardized to corresponding internal controls (sno202 [mouse] for miR‐29b expression and glyceraldehyde 3‐phosphate dehydrogenase for other genes). Values were expressed as the fold change (2^−(∆∆CT[microRNA]−∆∆CT[sno202])^). All experiments included at least four replicates per group.

### Statistical analysis

Statistical analysis was performed using SPSS 18.0 (SPSS Inc, Chicago, IL). Data are presented as mean ± standard error (SEM). Results are compared to age‐matched, littermate WT controls or scramble control treated, if not otherwise stated.

When comparing aortic diameter growth pattern with age between treatment groups, repeated measure analysis of variance (ANOVA) was used. One‐way ANOVA was utilized to determine differences between multiple treatment groups at each time point. Significance of individual differences was evaluated using the Bonferroni correction for multiple comparisons. Two‐tailed Student's *t*‐test for parametric data was used to compare groups. A value of *P* < 0.05 was considered statistically significant.

## Results

### Longitudinal progression of AS aortic aneurysms in the *Fbn1* ^C1039G/+^ Marfan mouse model

**Figure 1:**
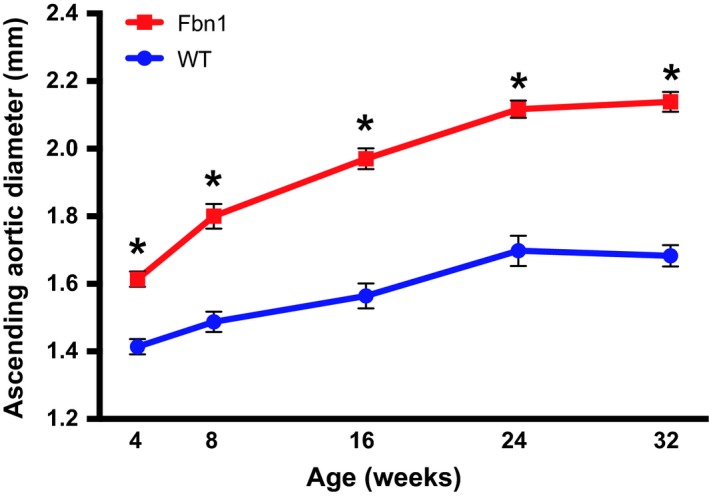
Long‐term development of AS aortic aneurysms in *Fbn1* ^C1039G/+^ mice. AS aortic diameter (mm) in *Fbn1* ^C1039G/+^ (squares) compared with littermate wild‐type control (circles) mice using transthoracic echocardiography (*n* = 8 at each time point, except *n* = 10 at 24 weeks). Results presented as mean ± SEM. *P < 0.0001.

AS aortic diameters in *Fbn1* ^C1039G/+^ and C57BL/6J WT littermate control mice were measured using TTE at 4, 8, 16, 24 and 32 weeks of age (Fig. 1). By 32 weeks, the aortic diameter in *Fbn1* ^C1039G/+^ mice reached 2.14 ± 0.03 mm in comparison to 1.68 ± 0.03 mm in WT controls (*P* < 0.001).

**Figure 2:**
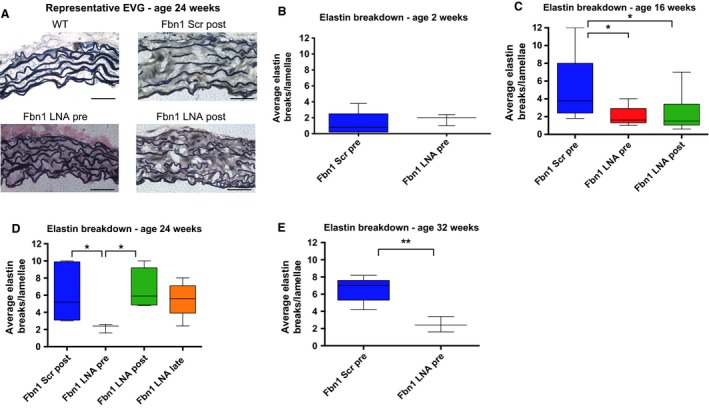
Increased elastin degradation in *Fbn1* ^C1039G/+^ mice and protective effect of miR‐29b inhibition against elastin breakdown. (A) Representative elastin histological staining of AS aorta from littermate wild‐type control (WT), postnatal scrambled control (Scr post), *Fbn1* ^C1039G/+^ prenatal LNA‐anti‐miR‐29b (LNA pre), and postnatal LNA‐anti‐miR‐29b (LNA post)‐treated mice at age 24 weeks. Scale bars represent 50 *μ*m. (B) Average number of elastic lamina breaks per lamellae in AS aorta (360‐degree) of *Fbn1* ^C1039G/+^ prenatal LNA‐anti‐miR‐29b (LNA pre; *n* = 5) or *Fbn1* ^C1039G/+^ prenatal scrambled control (Scr pre)‐treated mice at age 2 weeks (*n* = 5). (C) Average number of elastic lamina breaks per lamellae in AS aorta (360‐degree) of *Fbn1* ^C1039G/+^ prenatal scrambled control (Scr pre; *n* = 7), *Fbn1* ^C1039G/+^ prenatal LNA‐anti‐miR‐29b (LNA pre; *n* = 5) and *Fbn1* ^C1039G/+^ postnatal LNA‐anti‐miR‐29b (LNA post)‐treated mice at age 16 weeks (*n* = 6). (D) Average number of elastic lamina breaks per lamellae in AS aorta (360‐degree) of *Fbn1* ^C1039G/+^ postnatal scrambled control (Scr post; *n* = 5), *Fbn1* ^C1039G/+^ prenatal LNA‐anti‐miR‐29b (LNA pre; *n* = 3), *Fbn1* ^C1039G/+^ postnatal LNA‐anti‐miR‐29b (LNA post; *n* = 4) and *Fbn1* ^C1039G/+^ LNA‐anti‐miR‐29b–treated mice between 8 and 24 weeks (LNA 8–24 weeks; *n* = 5) at age 24 weeks. (E) Average number of elastic lamina breaks per lamellae in AS aorta (360‐degree) of scrambled‐control–treated (Scr pre; *n* = 5) mice or *Fbn1* ^C1039G/+^ prenatal LNA‐anti‐miR‐29b (LNA pre; *n* = 5) at age 32 weeks. Results presented as mean ± SEM. *P < 0.05. **P < 0.01.

Histological characterization with EVG staining revealed severe elastin fragmentation during aneurysm formation in *Fbn1* ^C1039G/+^ mice compared to WT controls (Fig. 2A). Although we did not detect any difference in elastin breakdown at age 2 weeks (Fig. 2B), elastin fragmentation was significantly elevated in *Fbn1* ^C1039G/+^ mice by 4 weeks, and persisted through 32 weeks (Fig. 2C–E).

**Figure 3:**
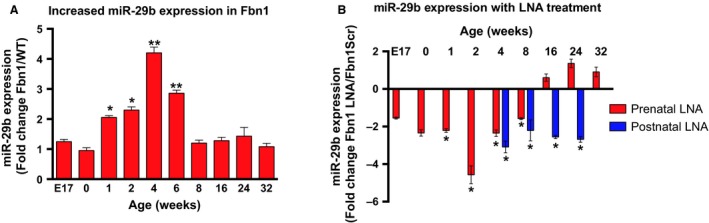
Increase in miR‐29b expression in *Fbn1* ^C1039G/+^ mice and miR‐29b suppression by locked nuclear acid (LNA) miR‐29b blockade in vivo. (A) Fold difference in miR‐29b expression by quantitative polymerase chain reaction (qPCR) in AS aorta of *Fbn1* ^C1039G/+^ compared to littermate wild‐type control (WT) mice at embryo day 17 (E17; *n* = 6), 0 (*n* = 5), 1 (*n* = 6), 2 (*n* = 7), 4 (*n* = 6), 6 (*n* = 4), 8 (*n* = 5), 16 (*n* = 8), 24 (*n* = 8), and 32 weeks of age (*n* = 8). B. miR‐29b expression level in AS aorta of *Fbn1* ^C1039G/+^ prenatal or postnatal LNA‐anti‐miR‐29b treatment (LNA pre or post) compared to *Fbn1* ^C1039G/+^ LNA scrambled mutated sequence control (Scr pre or post) at E17 (*n* = 3), 0 (*n* = 4), 1 (*n* = 7), 2 (*n* = 6), 4 (*n*−4 pre; *n* = 6 post), 8 (*n* = 5 pre; *n* = 4 post), 16 (*n* = 4 pre; *n* = 6 post), 24 (*n* = 3 pre; *n* = 4 post), and 32 (*n* = 4 pre) weeks of age. Results presented as mean ± SEM. *P < 0.05. **P < 0.01.

Previously, we reported that miR‐29b, a miRNA involved in ECM remodeling and apoptosis, was significantly upregulated in the *Fbn1* ^C1039G/+^ AS aorta beginning at 2 weeks, peaked by 4 weeks, then returned to baseline by 8 weeks (Merk et al. 2012). Although other investigators have reported significant changes in miR‐29b expression at later time points during both AS aortic and AAA development (Maegdefessel et al. 2012), no significant change in miR‐29b expression was detected in the *Fbn1* ^C1039G/+^ AS aorta at later time points (8–32 weeks) compared to WT (Fig. 3A).

### Early blockade of miR‐29b confers durable protection against aneurysm progression through 32 weeks

Although we have previously noted that aortic dilation can be prevented up to age 4 weeks in *Fbn1* ^C1039G/+^ mice when treated prenatally with LNA anti‐miR‐29b oligonucleotide inhibitor (Merk et al. 2012), it remains unknown whether miR‐29b blockade offers long‐term protection against aortic aneurysm formation. Moreover, the optimal treatment strategy for aneurysm prevention is not clear. Thus, in this study, *Fbn1* ^C1039G/+^ mice were treated with LNA‐anti‐miR‐29b inhibitor or scrambled‐control‐miR before aneurysms developed, either (1) as a single dose administered prenatally, or (2) postnatally every other week, starting at 2 weeks of age. Then, to determine if miR‐29b blockade was beneficial even after aneurysms develop, a third group of animals were treated every other week, starting at 8 weeks of age.

**Figure 4:**
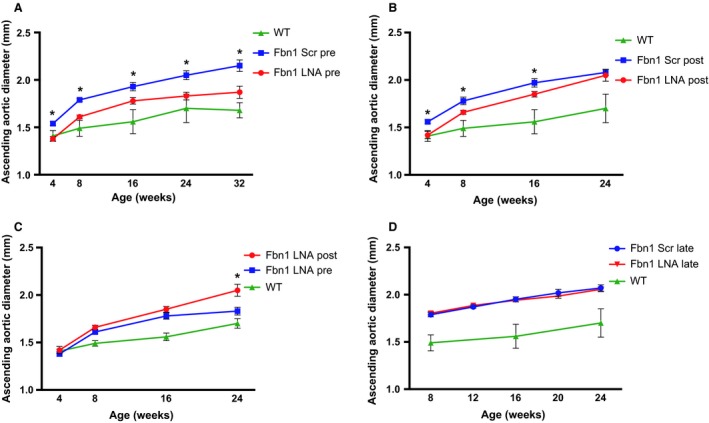
Reduction in AS aortic aneurysm formation and development by miR‐29b inhibition. (A) AS aortic diameter in littermate wild‐type control (WT) [green triangle], *Fbn1* ^C1039G/+^ prenatal LNA‐anti‐miR‐29b (LNA pre) [red circle], or *Fbn1* ^C1039G/+^ scrambled control (Scr pre) [blue square]‐treated mice using transthoracic echocardiography (*n* = 8–10, each group). *P < 0.05 for LNA pre versus Scr pre. (B) AS aortic diameter in littermate wild‐type control (WT) [green triangle], *Fbn1* ^C1039G/+^ postnatal LNA‐anti‐miR‐29b (LNA post) [red circle] or *Fbn1* ^C1039G/+^ scrambled control (Scr post) [blue square]‐treated mice using TTE;* n* = 8–10. *P < 0.05 for LNA post versus Scr post. (C) Comparison of AS aortic diameter between littermate wild‐type control (WT) [green triangle], prenatal (LNA pre) [blue square] or postnatal LNA‐anti‐miR‐29b (LNA post) [red circle]‐treated *Fbn1* ^C1039G/+^ mice (*n* = 8–10, each group). *P < 0.05 for LNA pre versus LNA post. (D) AS aortic diameter in littermate wild‐type control (WT) [green triangle], *Fbn1* ^C1039G/+^ LNA‐anti‐miR‐29b (red triangle) or scrambled control (blue circle) treated between age 8 and 24 weeks using TTE; (*n* = 4–6, each group). Results presented as mean ± SEM. *P < 0.05.

Both early treatment regimens with the LNA‐anti‐miR‐29b inhibitor significantly reduced miR‐29b expression levels as measured by quantitative polymerase chain reaction, compared to scrambled‐control–treated groups (Fig. 3B). In the prenatal treatment group, aortic dilation in LNA‐treated *Fbn1* ^C1039G/+^ mice compared to the scrambled‐control–treated mice was significantly reduced for up to 32 weeks, when the experiment was electively terminated (Fig. 4A). Surprisingly, continuous postnatal LNA‐inhibitor treatment was less effective than the prenatal regimen (single dose), despite superior miR‐29b reduction at later time points. When the treatment strategies were compared, aortic dilatation was not significantly different up to 16 weeks, however aortic dilation was significantly less in the prenatal treatment group at 24 weeks (Fig. 4B and C). Of note, although aortic dilatation was absent at age 4 weeks with both LNA‐treatment strategies, aneurysms eventually developed, although at a reduced rate. Moreover, aortic diameters in LNA‐treated animals remain greater than WT control after 4 weeks (Fig. 4A–D). These findings suggest that miR‐29b‐independent events may predominate in the progression of aneurysms at later time points.

To determine if miR‐29b blockade can slow aortic growth once aneurysms have already developed, we treated *Fbn1* ^C1039G/+^ mice with either LNA‐anti‐miR‐29b inhibitor or scrambled‐control‐miR every other week from 8 to 24 weeks. Aortic diameter was measured at 8, 12, 16, 20, and 24 weeks. We did not find a significant difference in aortic dimension between treatment and control groups (Fig. 4D). These results support our hypothesis that the molecular mechanisms that dictate late aneurysm growth are independent of miR‐29b expression. Taken together, miR‐29b suppression in early development is essential for long‐term reduction in aneurysm formation.

### miR‐29b blockade increases ECM and decreases MMP‐mediated elastin breakdown

**Figure 5:**
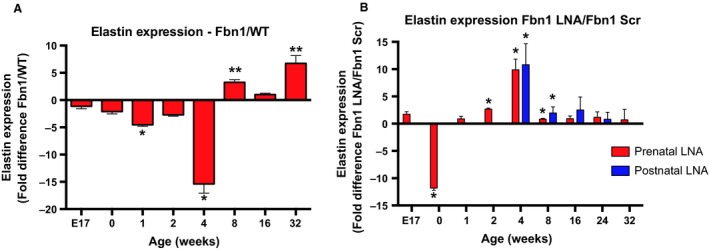
LNA treatment increases elastin gene expression at 4 weeks. (A) Fold difference in elastin (Eln) gene expression by quantitative polymerase chain reaction (qPCR) in AS aorta of *Fbn1* ^C1039G/+^ mice compared to littermate wild‐type controls at E17, 0, 1, 2, 4, 8, 16, 24, 32 weeks of age (*n* = 4–6, each group). Results presented as mean ± SEM. *P < 0.05. **P < 0.01. (B) Fold difference in Eln gene expression by qPCR in AS aorta of *Fbn1* ^C1039G/+^ prenatal anti‐miR‐29b LNA compared to *Fbn1* ^C1039G/+^ scrambled control (Scr pre) at E17 (*n* = 5), 0 (*n* = 6), 1 (*n* = 7), 2 (*n* = 5), 4 (*n* = 4), 8 (*n* = 6), 16 (*n* = 3), 24 (*n* = 3), and 32 (*n* = 7) weeks of age and *Fbn1* ^C1039G/+^ postnatal anti‐miR‐29b LNA compared to *Fbn1* ^C1039G/+^ scrambled control (Scr post)‐treated mice at 4 (*n* = 5), 8 *n* = 4), 16 (*n* = 3), and 24 (*n* = 5) weeks of age (*n* = 4–6, each group). Results presented as mean ± SEM. *P < 0.05. **P < 0.01.

We next aimed to define the mechanisms allowing for the superior efficacy of the prenatal miR‐29b blockade to reduce aneurysm formation long‐term. Correlating with a significant reduction in miR‐29b expression by 1 week of life, prenatal treatment increased *elastin* gene expression from baseline levels that were 15.4 ± 1.7‐fold lower than WT levels at 4 weeks of age (*P* = 0.04), to 2.6±0.2‐fold higher than scramble control levels (*P* = 0.04), by 2 weeks of age (Fig. 5A and B). In contrast, postnatal LNA miR‐29b blockade (beginning at 2 weeks of age) did not significantly increase *elastin* gene expression until 4 weeks of age. Of note, neither prenatal nor postnatal LNA miR‐29b blockade resulted in increased *elastin* gene expression past 8 weeks of age (Fig. 5). These results suggest that LNA miR‐29b blockade prevents an early impairment in elastogenesis in the *Fbn1* ^C1039G/+^ Marfan mouse model, correlating with the normal time period when aortic elastin synthesis occurs in mice (Davis 1995). Furthermore, prenatal treatment may be more beneficial for aneurysm reduction due to an earlier increase in *elastin* gene expression.

**Figure 6:**
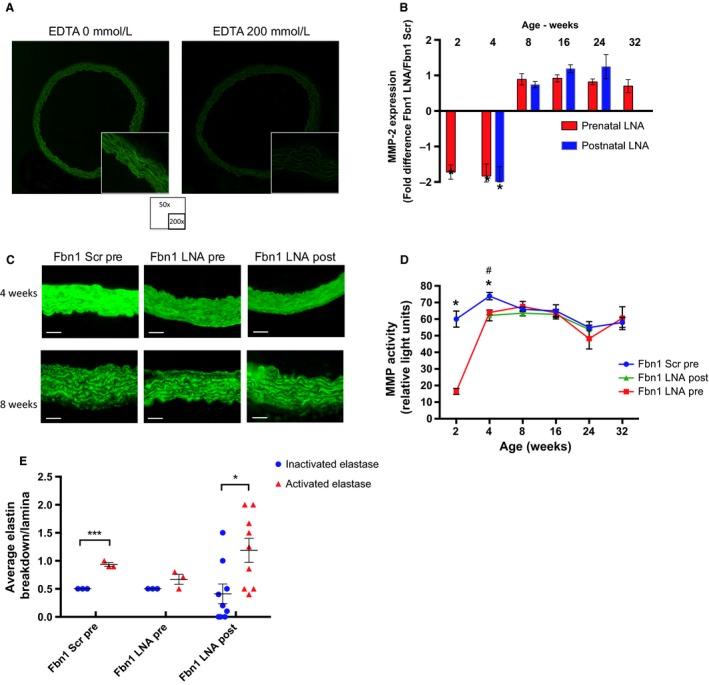
Upregulation of Matrix Metalloproteinase (Mmp) in *Fbn1* ^C1039G/+^ mice. (A) Elastase activity in AS aorta of 4‐week‐old *Fbn1* ^C1039G/+^ mice with or without ethylenediaminetetraacetic acid (EDTA), a pan‐Mmp inhibitor. Histology magnification at 50 and 200×. (B) Fold difference in Mmp gene expression by quantitative polymerase chain reaction (qPCR) in AS aorta of *Fbn1* ^C1039G/+^ prenatal LNA‐anti‐miR‐29b, *Fbn1* ^C1039G/+^ postnatal LNA‐anti‐miR‐29b, or *Fbn1* ^C1039G/+^ scrambled‐control–treated mice at 2 (*n* = 6 pre), 4 (*n* = 4 pre; *n* = 4 post), 8 (*n* = 5 pre; *n* = 5 post), 16 (*n* = 3 pre; *n* = 6 post), and 32 (*n* = 9 pre) weeks of age. Results presented as mean ± SEM. *P < 0.05. (C) Representative in situ zymography of AS aortas of *Fbn1* ^C1039G/+^ Scr pre, LNA pre, and LNA post at 4 and 8 weeks. (D) Mmp activity in AS aorta of *Fbn1* ^C1039G/+^ prenatal LNA‐anti‐miR‐29b (LNA pre) (red square), postnatal LNA‐anti‐miR‐29b (LNA post) (green triangle), and *Fbn1* ^C1039G/+^ prenatal scrambled control treated (Scr pre) (blue circle) at 2, 4, 8, 16, 24, and 32 weeks of age (*n* = 3 at each time point, except *n* = 4 at 4 weeks). Data are expressed as percentage compared with 4‐week‐old *Fbn1* ^C1039G/+^ prenatally scrambled control treated values. *P < 0.05 for LNA pre versus Scr pre. ^#^ *P* < 0.05 for LNA post versus Scr pre. (E) Discontinuity score of the AS aorta treated with either inactivated or activated elastase in 4‐week‐old *Fbn1* ^C1039G/+^ prenatal scrambled control (Scr pre; *n* = 5), prenatal LNA‐anti‐miR‐29b (LNA pre; *n* = 5), and postnatal LNA‐anti‐miR‐29b (LNA post) (*n* = 10). The following scoring system was utilized: (0: normal, 1: 1–2 fold increase in elastic lamellar fenestration size; 2: 2–4 fold increase in elastic lamellar fenestration size). Results presented as mean ± SD ***P < 0.001, *P < 0.05.

In addition to restoring early elastogenesis, both pre‐ and postnatal miR‐29b blockade also decreased elastin fragmentation, although again prenatal treatment appears more beneficial long‐term (Fig. 2C–E). Elastin degradation can be mediated by a variety of enzymes, including MMPs, serine proteinases, and cysteine proteinases (Benjamin 2001; Liu et al. 2004; Wagsater et al. 2012; Werb et al. 1982). We elected to focus on MMPs, given their implication in numerous other models of aortic aneurysms (Benjamin 2001; Daugherty and Cassis 2004; Ikonomidis et al. 2006; Maegdefessel et al. 2012; Merk et al. 2012) and since they are downstream targets of miR‐29b (Chen et al. 2011). Of note, in situ zymography with DQ‐gelatin utilized to quantify MMP activity, may also reflect other contributing proteases. To demonstrate that the elastin breakdown observed in this Marfan mouse model was due to enhanced MMP activity, non‐MMP activity was measured in 4 weeks *Fbn1* ^C1039G/+^ AS aortic wall specimens treated with EDTA, a pan‐MMP inhibitor. No significant elastase activity was detected in the EDTA‐treated specimens (Fig. 6A), suggesting that elastin fragmentation present at 4 weeks can be attributed to MMPs. Importantly, we cannot exclude the possibility that EDTA cross‐reacts and blocks other matrix degrading enzymes.

Prenatal treatment with LNA anti‐miR‐29b decreased *Mmp2* gene expression in *Fbn1* ^C1039G/+^ mice at 2 and 4 weeks, and postnatal treatment decreased *Mmp2* gene expression at 4 weeks. Neither treatment strategy resulted in changes in *Mmp2* gene expression at 8, 16, 24, or 32 weeks (Fig. 6B). In situ zymography with DQ gelatin was used to quantify Mmp2 and ‐9 activity levels (Mook et al. 2003). Corresponding with enhanced elastin breakdown detected in Marfan AS aortic aneurysms, Mmp2 and ‐9 activity levels were increased in *Fbn1* ^C1039G/+^ scrambled control mice compared to WT control at all time points, peaking by 4 weeks (Fig. 6C and D). Significantly reduced Mmp activity levels are measured as early as 2 weeks in the prenatal treatment group only, whereas both LNA blockade treatment strategies significantly reduced Mmp activity levels at 4 weeks, which returned to baseline by 8 weeks. The superior results detected with prenatal treatment may be beneficial due to earlier Mmp blockade.

Interestingly, although Mmp activity increased in both groups by 8 weeks, elastin breakdown remained reduced only in the prenatal treatment animals through 32 weeks (Fig. 2E). These data suggested that prenatal inhibition of miR‐29b during early aortic wall development may confer decreased susceptibility for aortic wall elastin breakdown. To test this hypothesis, an elastin susceptibility test was performed on aortic wall specimens from 2‐week‐old *Fbn1* ^C1039G/+^ mice treated with either (a) prenatal LNA‐anti‐miR‐29b inhibitor, (b) prenatal scrambled‐control‐miR, (c) postnatal LNA‐anti‐miR‐29b inhibitor, or (d) postnatal scrambled‐control‐miR. No difference in aortic wall elastin discontinuity was detected when specimens were incubated with inactivated elastase. Notably, while activated elastase increased elastin fragmentation in *Fbn1* ^C1039G/+^ mice treated with either postnatal treatment or scrambled control, the elastin breakdown was significantly reduced in *Fbn1* ^C1039G/+^ mice treated prenatally with the LNA‐anti‐miR‐29b inhibitor (Fig. 6E).

### Increased aortic wall collagen deposition following miR‐29b blockade is not protective

**Figure 7:**
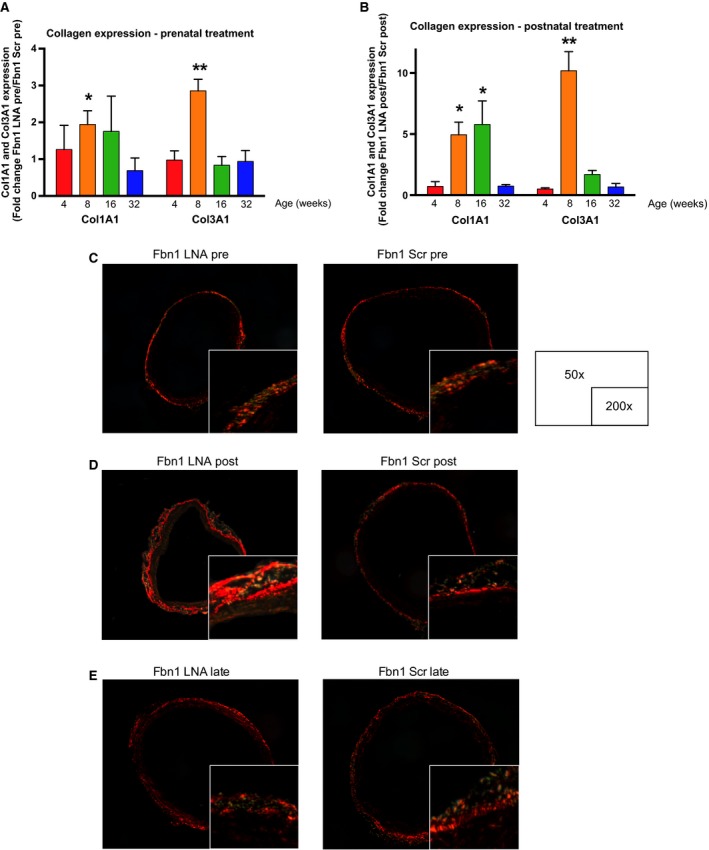
Collagen type I and III increased by miR‐29b inhibition. (A) Fold difference in ColIA1 and ColIIIA1 gene expression by quantitative polymerase chain reaction (qPCR) in AS aorta (AS) of *Fbn1* ^C1039G/+^ prenatal LNA‐anti‐miR‐29b (LNA pre) versus *Fbn1* ^C1039G/+^ scrambled control (Scr pre)‐treated mice at 4 (*n* = 3), 8 (*n* = 4), 16 (*n* = 3), and 32 (*n* = 7) weeks of age. (B) Fold difference in ColIA1and ColIIIA1 gene expression by qPCR in *Fbn1* ^C1039G/+^ AS postnatal LNA‐anti‐miR‐29b (LNA post) versus scrambled control (Scr post)‐treated mice at 4 (*n* = 3), 8 (*n* = 5), 16 (*n* = 4), and 32 (*n* = 6) weeks of age. (C) Representative polarized light microscopy of picrosirius red staining of AS aorta in *Fbn1* ^C1039G/+^ prenatal LNA‐anti‐miR‐29b (LNA pre) or scrambled control (Scr pre)‐treated mice at 24 weeks of age. (D) Representative polarized light microscopy of picrosirius red staining of *Fbn1* ^C1039G/+^ AS aorta postnatal LNA‐anti‐miR‐29b (LNA post) or scrambled control (Scr post)‐treated mice at 24 weeks of age. (E) Representative polarized light microscopy of picrosirius red staining of *Fbn1* ^C1039G/+^ AS aorta late LNA‐anti‐miR‐29b (LNA late) or scrambled control (Scr late)‐treated mice at 24 weeks of age. Results presented as mean ± SEM. *P < 0.05. **P < 0.01.

miR‐29b negatively regulates the gene expression of *collagen types I and III*, major ECM components within the aortic wall (Judge and Dietz 2005). Correlating with reduced miR‐29b levels, *collagens types I and III* gene expression was enhanced in the prenatal group at 8 weeks (*collagen type I*, 1.94±0.37‐fold, *P* = 0.047; *collagen type III*, 2.85 ± 0.31‐fold, *P* < 0.001), and at both 8 and 16 weeks in the postnatal group (*collagen type I*, 4.95 ± 1.03‐fold, *P* = 0.019 and 7.23 ± 2.32‐fold, *P* = 0.045, respectively; and *collagen type III*, 10.18 ± 1.57‐fold, *P* = 0.004 and 1.69 ± 0.34‐fold, *P* = 0.176, respectively) (Fig. 7A and B). Although *collagen* gene expression was elevated, histological staining with picrosirius red revealed that prenatal LNA anti‐miR‐29b inhibitor did not change aortic wall collagen deposition (prenatal: 40.7 ± 2.5 pixel intensity vs. scrambled control: 36.6 ± 0.7 pixel intensity), even though aneurysms are reduced through age 32 weeks (Fig. 7C). Interestingly, repeated postnatal doses of LNA anti‐miR‐29b inhibitor resulted in enhanced aortic wall collagen deposition (postnatal: 52.8 ± 3.1 pixel intensity vs. scrambled control: 36.6 ± 0.7 pixel intensity, *P* = 0.01), yet no significant difference in aneurysm formation was noted at 24 weeks (Fig. 7D). Finally, late LNA treatment starting at 8 weeks did not increase aortic wall collagen deposition (late: 40.7 ± 3.7 pixel intensity) (Fig. 7E). Of note, picrosirius staining color changes do not reflect composition of collagen fibers (Lattouf et al. 2014).

Because LNA miR‐29b blockade is administered systemically, the off‐target effects were also examined. Collagen deposition and fibrosis was not increased in the heart or kidney, regardless of treatment strategy. In the postnatal treatment group only, collagen deposition was slightly increased within the liver.

## Discussion

Studies have reported that mutations within the TGF‐*β* signaling pathway can result in a number of inheritable connective tissue disorders, including the increased systemic TGF‐*β* observed in Marfan syndrome, the mutated TGF‐*β* receptors seen in Loeys‐Dietz syndrome, or the altered downstream TGF‐*β* canonical signaling noted in Smad3 deficiency (Braverman 2013; Guo et al. 2006; Habashi et al. 2006; Judge and Dietz 2005; Neptune et al. 2003). While progress has been made in understanding aneurysm formation during the last decade, the molecular mechanisms downstream of TGF‐*β* still remain unknown. For example, in Marfan syndrome, why aneurysms localize exclusively to the aortic root despite enhanced systemic TGF‐*β* signaling is unexplained. Investigating the pathways that participate in ECM degeneration in the aortic root may lead to novel, anatomically‐directed, targeted therapies to prevent or treat aneurysm formation in Marfan syndrome. In this study, using the *Fbn1* ^C1039G/+^ Marfan mouse model, we illustrate that (1) miR‐29b expression is only transiently increased in the AS aorta; (2) miR‐29b blockade with an oligonucleotide inhibitor slows aneurysm growth long‐term; (3) a single dose of prenatal miR‐29b inhibitor is superior to a continuous postnatal regimen; and (4) the beneficial effects of miR‐29b blockade seem most closely associated with an increase in elastin production and stability, and a decrease in Mmp activity.

The aorta is a heterogeneous structure composed of three layers, the intima, media and adventitia. Lineage studies have shown that vascular smooth muscle cells (SMC) in the different anatomic segments of the aorta have distinct embryological origins (Sinha et al. 2014). Specifically, the aortic root is derived from the second heart field (Waldo et al. 2005). The ascending aorta and arch to the ligamentum arteriosum originate from neural crest cells (Waldo et al. 2005). The descending/abdominal aortas develop from paraxial mesoderm. Recent studies have suggested that the diversity of SMC origin may explain site specific location of various vascular diseases, including atherosclerosis, aortic dissection and aneurysm development (Milewicz et al. 2008). Corroborating this theory, we found that miR‐29b is increased during early postnatal development exclusively in SMC derived from the *Fbn1* ^C1039G/+^ AS aorta (Merk et al. 2012), correlating with aneurysm location. miR‐29b functions to regulate hundreds of genes, including those involved in SMC apoptosis, elastogenesis, fibrillin deposition, extracellular matrix breakdown, and collagen deposition (Maegdefessel et al. 2012; Merk et al. 2012). While TGF‐*β* appears to regulate miR‐29b transcription in aortic SMC (Maegdefessel et al. 2012; Merk et al. 2012; Milewicz 2012), how TGF‐*β* increases miR‐29b expression in a manner that is temporally and spatially restricted to the AS aorta during early postnatal life, is not clear. It is possible that SMC that originate from the AS aorta respond differently to TGF‐*β* compared to SMC from the descending aorta in this mouse model. miR‐29b is regulated at the transcriptional and posttranscriptional levels (Mott et al. 2010). Putative repressors of miR‐29b transcription include *c‐Myc, Hedgehog, Smad3, Ying‐Yang1, TCF/LEF,* and *NF‐κB* (Javelaud et al. 2011; Johnson et al. 2011). We have previously shown that the negative regulators of miR‐29b, *c‐Myc and NF‐κB* are reduced in the AS aorta only, leading to increased miR‐29b expression (Merk et al. 2012). A more complete understanding of miR‐29b transcriptional regulation in response to TGF‐*β* may help identify why the transient, but pathologic increase in miR‐29b occurs uniquely in the AS aorta of *Fbn1* ^C1039G/+^ mice, and serve to identify novel, anatomically directed therapies.

One of the key findings of this study was that a single, prenatal dose of the miR‐29b inhibitor was more effective than continuous postnatal administration of the inhibitor. Thus, we aimed to define the mechanism(s) allowing for this superior reduction in aneurysm formation. Elastin is synthesized by SMC as the monomer, tropoelastin, and represents the dominant arterial wall ECM protein. Following post‐translational modification, tropoelastin is cross‐linked and organized into elastin polymers, forming alternating concentric rings of elastic lamella and SMC. Although initially thought to play mostly a structural role, there is growing evidence that elastin may be important during arterial wall morphogenesis for vascular SMC stabilization (Brooke et al. 2003). Using elastin deficient mice (*Eln* ^−/−^), which are lethal due to an occlusive vascular fibrocellular pathology (Li et al. 1997), investigators have illustrated that elastin is important for SMC phenotypic modulation, proliferation and migration via G‐protein coupled signaling pathways (Karnik et al. 2003; Li et al. 1998). We report here that elastin levels in the aortic media are 15‐fold lower in Marfan *Fbn1* ^C1039G/+^ mice at 4 weeks than WT controls. Perhaps because of drug administration timing, only prenatal miR‐29b blockade reversed this early impairment in elastogenesis, resulting in aortic media elastin levels that were 2.6‐fold higher than those found in *Fbn1* ^C1039G/+^ scramble controls. In addition, both prenatal and postnatal miR‐29b blockade reduce Mmp induced elastin breakdown. Interestingly, prenatal treatment resulted in more durable decreases in elastin breakdown than postnatal treatment, despite similar levels of Mmp activity by 8 weeks in both treatment groups. Although the advantage may be in part due to earlier Mmp blockade, prenatal treatment also seems to confer a developmental benefit to elastin which makes it less susceptible to elastase breakdown. Taken together, although not directly tested in this study, our data suggest that prenatal miR‐29b blockade is the superior treatment strategy as it results in the stabilization of elastin levels during embryologic development, which appears to be important temporally for normal arterial wall morphogenesis. This hypothesis has obvious clinical relevance, suggesting that novel therapies directed at aneurym prevention in Marfan syndrome may need to be initiated in utero.

The role of miR‐29b in the development of abdominal aneurysms has been the focus of previous studies (Milewicz 2012). Maegdefessel et al. (2012) reported that reduced miR‐29b expression is a late compensatory response during AAA development in both angiotensin II treated Apoe^−/−^ and porcine pancreatic elastase AAA mouse models, resulting in enhanced collagen deposition, fibrosis and decreased aneurysm growth. In this study, enhanced aortic wall collagen deposition in the postnatal treatment group did not render any significant protection during AS aneurysm formation. Possible mechanisms explaining this disparity include: (1) differences in the experimental model system; (2) differences in the animal age/timing of miR‐29b expression; and (3) differences in aortic segment embryologic origin.

Crosas‐Molist et al. (2015) detected enhanced collagen deposition in dilated human Marfan aortic specimens compared to non‐dilated and healthy aortas. Whether this collagen deposition accelerates aneurysm progression or represents a beneficial response to reinforce the aortic wall hemodynamic stress remains controversial. Several studies have investigated aortic wall hemodynamic indices in Marfan syndrome, including wall compliance, wall stiffness index, and distensibility (Adams et al. 1995; Groenink et al. 1998; Savolainen et al. 1992). Baumgartner et al. (2005) found that high aortic wall distensibility was a favorable prognostic indicator, whereas reduced elasticity was predictive of aortic wall abnormalities in Marfan syndrome patients. Therefore, in our model system, the beneficial effects of continuous postnatal blockade may have been counterbalanced by the deleterious effects of reduced aortic wall elasticity (increased collagen deposition).

In conclusion, the current gap in knowledge surrounding the mechanisms leading to aortic aneurysms in Marfan syndrome has precluded the development of novel therapies to prevent aneurysm formation. This fact is highlighted by the disappointing absence of any therapeutic benefit aimed to reduce TGF‐*β* signaling in young Marfan patients in two recent randomized clinical trials (Lacro et al. 2014; Milleron et al. 2015). Here, we report that systemic miR‐29b inhibition reduces Marfan aneurysm development long‐term and provides new targets for innovative preventive strategies. Slowing aneurysm growth in Marfan patients is clinically beneficial, even if simply delaying surgical intervention. Because investigators have reported that miR‐29a levels were related to aortic size in human thoracic aneurysms (Jones et al. 2011), we believe miR‐29 inhibition may be applicable to treating other thoracic aortic aneurysms. Intriguingly, in this animal model, drug administration during aortic wall embryologic development seems fundamental; therefore, this newly described finding may serve as the foundation that could shift our current thinking as to the optimal timing of medical intervention in order to most effectively prevent or slow aneurysm development in patients with Marfan syndrome.

## Limitations

In the prenatal treatment group, *Fbn1* ^C1039G/+^ mice were measured up to 32 weeks, when the experiment was electively terminated. Longer follow‐up would be beneficial to determine the exact efficacy of early miR‐29b blockade. Importantly, because aneurysms eventually develop, miR‐29b‐independent events may predominate in the progression of aneurysms at later time points. In addition, although we report here that prenatal miR‐29b blockade is potentially beneficial via protection of the elastin architecture during early aortic wall development in mice, we cannot rule out the potential detrimental effects during embryogenesis.

## Conflict of Interest

Authors have nothing to disclose with regard to commercial support.
